# Triple-doped KMnF_3_:Yb^3+^/Er^3+^/Tm^3+^ nanocubes: four-color upconversion emissions with strong red and near-infrared bands

**DOI:** 10.1038/srep17088

**Published:** 2015-11-26

**Authors:** Hao Wang, Xiaodong Hong, Renlu Han, Junhui Shi, Zongjun Liu, Shujuan Liu, You Wang, Yang Gan

**Affiliations:** 1 School of Materials Science and Engineering, Harbin Institute of Technology, Harbin 150001. P. R. China; 2 School of Chemical Engineering and Technology, Harbin Institute of Technology, Harbin 150001. P. R. China; 3 College of Materials Science and Engineering, Liaoning Technical University, Fuxin city 123000. P. R. China

## Abstract

Triple-doped (Yb^3+^/Er^3+^/Tm^3+^) KMnF_3_ nanocubes with uniform sizes of 250 nm were synthesized by a facile hydrothermal route using the oleic acid as the capping agent. It was found that these nanocubes can simultaneously exhibited four-color (blue, green, red and NIR) upconversion emissions under a single 980 nm near-infrared (NIR) laser excitation, which should have potential multicolor *in vivo* imaging applications. Specifically, the red (660 nm) and NIR (800 nm) peaks, known as two “optical windows” for imaging biological tissues, were strong. The spectral and pump analyses indicated the two-photon processes were responsible for the both red and NIR emissions.

Upconversion nano-particles (UCNPs) have the ability to convert lower energy (near-infrared (NIR) or infrared (IR)) radiation into high-energy radiation (ultraviolet or visible) via multiphoton absorption and energy transfer (ET) processes^1^, which are promising for applications in optical bioimaging^2^,^3^, biodetection^4^,^5^, clinical diagnosis^6^, three-dimensional display technologies^7^, photocatalysis^8^, as well as solar cells^9^,^10^,^11^,^12^,^13^. To date, the rather popular UCNPs systems for biomedical imaging applications are mainly based on Er^3+^ and Tm^3+^ ions sensitized by Yb^3+^ ions with visible and near-infrared emissions^14^. The emissions of UCNPs in the red (660 nm, Er^3+^/Yb^3+^) and NIR regions (800 nm, Tm^3+^/Yb^3+^) are known as the two “optical windows” for imaging biological tissues^15^,^16^,^17^. The red emission is suitable for *in vitro* imaging because the images can be observed by naked eyes. *In vivo* imaging prefers NIR-to-NIR emissions, allowing certain penetration depth for inspection. For the red emissions, Bai *et al.*^15^ and Tian *et al.*^16^ showed that varying Yb^3+^ concentration or doping Mn^2+^ ions into the NaYF_4_ matrix were effective for red luminescence enhancement. For the NIR emissions, Chen *et al.*^18^ reported that the UC NIR emission at 800 nm was increased by 43 times in NaYF_4_:Yb^3+^/Tm^3+^ nanoparticles by heavily doping with Yb^3+^ ions. Recently, Liu group^19^ successfully prepared KMnF_3_ nanocrystals codoped with Yb^3+^/Er^3+^ or Yb^3+^/Tm^3+^ ions and found them showing substantially higher red and NIR emission intensity than that of the rare-earth doped NaYF_4_ nanocrystals. In general, most of the conventional imaging methods are monochrome and only able to detect one contrast agent at a time, limiting us to single parametric data. However, the unique properties of multicolor emissions, photostability, high penetration depth, and low photo damaging in principle enable UC materials to act as multi-color imaging probes for biomedical applications. In the pioneer work of multicolor imaging *in vivo,* Kobayashi *et al.*^20^ employed a polyamidoamine dendrimer platform linked to five dye molecules as different optical probes, permitting five-color optical imaging using a multiple-excitation spectrally resolved fluorescence imaging technique. However, simultaneously providing multicolor excitation lights and guarantee their penetration depths which are determined by the nature of light color itself are difficult, and thus it limits the practical applications of multicolor *in vivo* imaging.

Due to multicolor emission characteristics of UCNPs, simultaneous detection of multiple analytes or optical probes in a complex sample should be feasible if appropriate UCNPs were prepared and used. Ideally, *in vivo* multicolor tissue characterization^21^ relies on: (1) the identification of multiple targets; (2) target-specific optical probes with distinct fluorescent properties; and (3) effective real-time multicolor optical cameras that permit accurate unmixing of different fluorescent probes with a single NIR excitation *in vivo*. The present rare-earth doped nanocrystals are generally not suitable for multiplexing biodetection, due to their limited number of colors. It is therefore necessary to develop UCNPs with multicolor fluorescence emissions under NIR excitation at the same wavelength. Along this line, several recent studies focused on multicolor UC emission with a more boarder spectrum of color output by using different host/activator combinations. Rantanen *et al.*^22^ demonstrated simultaneous detection of two analytes using UC donors with multipeak emission characteristics. Nann *et al.*^23^ reported the preparation of complex colloidal UCNPs systems and observation of the four-color (blue, green, red and NIR) emissions. They synthesized four different types of UCNPs by doping NaYbF_4_ with different rare-earth ions, and thus obtain a four-color UC emission system for the potential multiplexing analysis by mixing these UCNPs. Jiang *et al.*^24^ prepared core-shell structured nanoparticles with UCNP core and dye-doped silica shell to enable multi-color emissions for multiplex bioassays.

Herein, we developed a facile hydrothermal strategy to obtain multicolor emissions by preparing tri-doped KMnF_3_:Yb^3+^/Er^3+^/Tm^3+^. The as-prepared UCNPs exhibit four-color (blue, green, red and NIR) UC emissions upon a single excitation at 980 nm, which should have a potential use in multicolor *in vivo* imaging for simultaneously providing multi-color excitation lights with deep imaging depth. In addition, UCNPs are a promising candidate to harvest NIR sunlight and improve the power conversion efficiency of solar cells, i.e., dye sensitized solar cell (DSSC)^25^. As some DSSC are designed based on the simultaneous adsorption of different dyes which have different absorption bands, the developed UCNPs with multi-color emission bands matching the absorption bands of dyes may have a potential to improve overall absorption efficiency^12^.

## Results and Discussion

**Figure 1:**
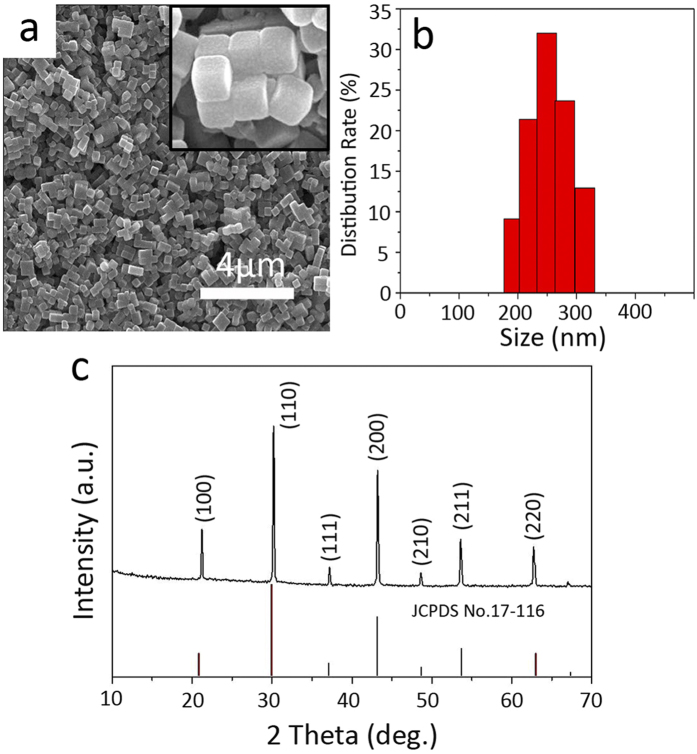
(**a**) SEM images of the as-synthesized KMnF_3_:20% Yb^3+^, 2% Er^3+^, 2% Tm^3+^ nanocubes. (**b**) Size distribution of KMnF_3_:20% Yb^3+^, 2% Er^3+^, 2% Tm^3+^ nanocubes. (**c**) XRD patterns of KMnF_3_:20% Yb^3+^, 2% Er^3+^, 2% Tm^3+^ nanocubes.

Figure 1a is a typical SEM image of as-prepared KMnF_3_:20%Yb^3+^/2%Er^3+^/2%Tm^3+^ UCNPs and Fig. 1b shows the average size distribution of the samples corresponding to those in Fig. 1a. It can be seen that the UCNPs are well dispersed and exhibit uniform nanocube shape with an average size of 250 nm. The crystal structures and the phase purity of the as-prepared tri-doped KMnF_3_ nanocubes were examined by the X-ray diffraction (XRD) analysis (see Fig. 1c). All peaks are sharp and match well with the standard JCPDS No.17-0116 of KMnF_3_, indicating high phase purity and crystallinity of obtained samples.

**Figure 2:**
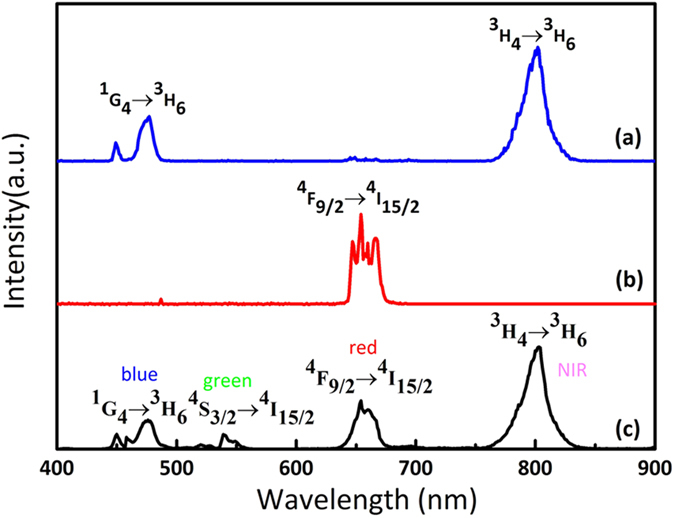
Calibrated UC emission spectra of KMnF_3_ samples under the excitation of a 980 nm. (**a**) Doped with 2 mol % Tm^3+^ and 20 mol % Yb^3+^, (**b**) doped with 2 mol % Er^3+^ and 20 mol % Yb^3+^, and (**c**) doped with 2 mol % Tm^3+^, 2 mol % Er^3+^ and 20 mol % Yb^3+^.

Typical UC emission spectra for various samples under diode laser excitation of 980 nm are shown in Fig. 2. KMnF_3_:Yb^3+^/Tm^3+^ samples show one blue emission band at 476 nm and one NIR band at 800 nm (Fig. 2a), corresponding to the ^1^*G*_4_ (Tm^3+^) → ^3^*H*_*6*_ (Tm^3+^), and ^3^*H*_4_ (Tm^3+^) → ^3^*H*_*6*_ (Tm^3+^) transitions of Tm^3+^ ions, respectively. KMnF_3_:20%Yb^3+^/2%Er^3+^ samples show only a single red emission at 660 nm corresponds to the ^4^*F*_9/2_ → ^4^*I*_15/2_ transitions of Er^3+^ ions (Fig. 2b). Very interestingly, tri-doped KMnF_3_:Yb^3+^/Er^3+^/Tm^3+^ nanocubes exhibits four-colored bands (Fig. 2c). Noteworthy that, besides three emission bands of 476 nm, 800 nm owing to Tm^3+^ ions and the 660 nm band owing to Er^3+^ ions are all preserved in the spectra, a new green UC emission centered at 540 nm is also observed at the same time.

Firstly, appearance of the new 540 nm green UC emission can be explained as follows. Usually, the single red UC emission (660 nm) is observed for Yb^3+^/Er^3+^ codoped KMnF_3_ samples. However, for tri-doped KMnF_3_:Yb^3+^/Er^3+^/Tm^3+^ samples, owing to the coexistence of Er^3+^ and Tm^3+^, the cross relaxation ^3^*F*_4_ (Tm) + ^4^*F*_9/2_ (Er) → ^1^*G*_4_ (Tm) + ^4^*I*_15/2_ (Er) between Tm^3+^ and Er^3+^ ions in KMnF_3_ may cause decreases in population of ^4^*F*_9/2_ state and increases in population of ^1^*G*_4_ state^26^. The green emission is thus generated through the ^2^*H*_11/2_/^4^*S*_3/2_ → ^4^*I*_15/2_ transition of Er^3+^ ions as explained in detail below.

**Figure 3:**
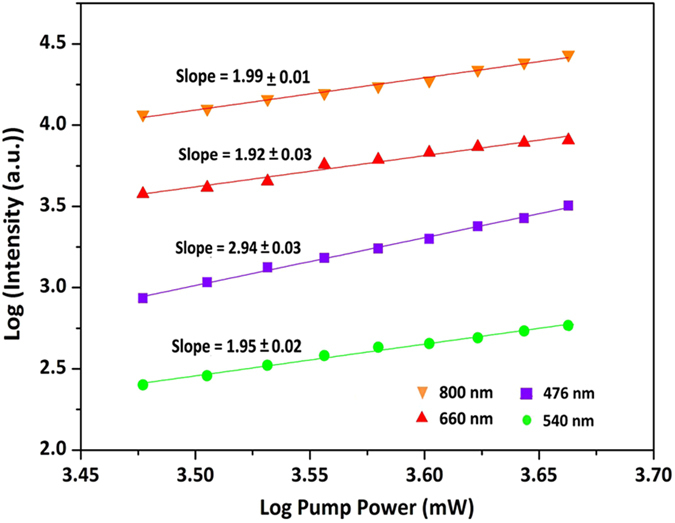
Logarithmic plots of the intensity of each upconversion band in Fig. 2c versus the excitation density in the dispersed KMnF_3_ nanocubes tridoped with 2 mol % Tm^3+^, 2 mol % Er^3+^ and 20 mol % Yb^3+^. The initial input power employed for the measurement is 2W.

Secondly, UC emission intensity (*I*) was further measured as a function of laser power (*P*) (Fig. 3) to explore the UC mechanism of Yb^3+^, Tm^3+^, and Er^3+^ ions in KMnF_3_ matrix. Because *I*_*UC*_ ∝ *P*^*n*^ holds for the unsaturated UC process, where *n* is the number of pump photons absorbed per upconverted photons emitted^27^, the value of *n* can thus be determined to be the slope after linearly fitting the I-P data in a double logarithmic plot. For the tri-doped KMnF_3_:Yb^3+^/Er^3+^/Tm^3+^ sample, the obtained *n* values are 2.94, 1.95, 1.92, and 1.99 respectively for the UC emission peaks at 476 nm (blue), 540 nm (green), 660 nm (red), and 800 nm (NIR). Therefore, it can be deduced that the three-photon process is responsible for blue UC emission, two-photon process is responsible for green red and 800 nm UC emissions.

**Figure 4:**
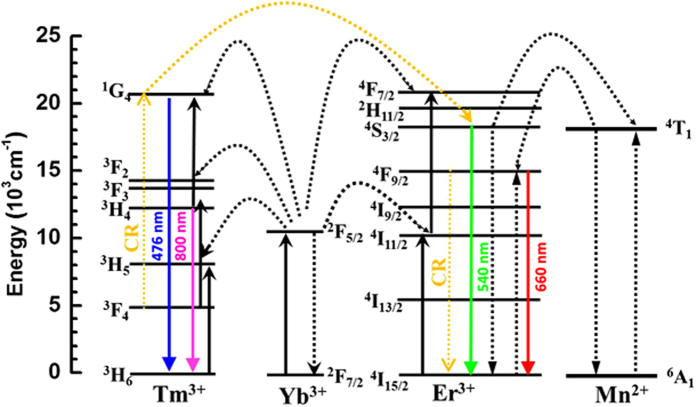
Schematic of the energy level diagram for the Er^3+^, Tm^3+^, and Yb^3+^ ions as well as the proposed UC mechanisms to explain the blue, green, red and NIR UC emissions. CR = Cross relaxation.

At last, the overall UC emission mechanism and population process in rare-earth doped KMnF_3_ is schematically illustrated in Fig. 4. Upon excitation at 980 nm, the red UC emission (660 nm) can be ascribed to nonradiative energy transfer from the ^4^*S*_3/2_ levels of Er^3+^ to the ^4^*T*_1_ level of Mn^2+^, followed by the falling-back transition to the ^4^*F*_9/2_ level of Er^3+^ and the ^4^*F*_9/2_ to ^4^*I*_15/2_ transition.

It would be interesting to have a closer look at the role of Mn^2+^ played in the multi-photon excited mechanism, based on the literature findings, for both double-doped KMnF_3_:Yb/Er system and triple-doped KMnF_3_:Yb/Er/Tm system. For the simpler double-doped KMnF_3_:Yb/Er system, it is accepted that Mn^2+^ ions play the important role in the single-band UC emission (the complete disappearance of 540 nm green emission and appearance of only 660 nm red emission). According to the literature, close proximity and excellent overlap of energy levels of the Mn^2+^ and Er^3+^ ions in the host lattices cause very efficient nonradiative energy transfer from the ^2^H_11/2_ and ^4^S_3/2_ levels of Er^3+^ to the ^4^T_1_ level of Mn^2+ ^16,28. And this nonradiative energy transfer process is followed by the back-energy transfer to the ^4^F_9/2_ level of Er^3+^, thus leading to only 660 nm red emission. The mechanism is illustrated in the right part of Fig. 4 where only three Yb^3+^, Mn^2+^ and Er^3+^ ions are involved. On the other hand, for the more complex triple-doped KMnF_3_:Yb/Er/Tm system, as illustrated in Fig. 4 where all four Yb^3+^, Mn^2+^, Er^3+^ and Tm^3+^ ions are involved, reappearance of 540 nm green emission is due to the additional resonant cross relaxation process between Er^3+^ and Tm^3+^ ions:^3^F_4_ (Tm^3+^) + ^4^F_9/2_ (Er^3+^) → ^1^G_4_ (Tm^3+^) + ^4^I_15/2_ (Er^3+^). This process causes the population of ^1^G_4_ state of Tm^3+^ ions and depopulation of ^4^F_9/2_ state of Er^3+^ ions. Because the energy level of ^1^G_4_ state (Tm^3+^) equals to that of ^4^F_7/2_, photons loose fraction of energy in ^4^F_7/2_ (Er^3+^) and drop to ^2^H_11/2_/^4^S_3/2_ (Er^3+^) state through the multiphonon assisted relaxations, and finally leading to 540 nm green emission.

For the blue (476 nm) and NIR (800 nm) emissions, the energy transfer from the first Yb^3+^ → Tm^3+^ excites the ^3^*H*_6_ → ^3^*H*_5_ transition, at the same time the redundant energy dissipated by phonons. Then, the Tm^3+^ ion is firstly relaxes to the lower ^3^*F*_4_ state and further promoted to the ^3^*F*_2_,_3_ state through a continuous Yb^3+^ → Tm^3+^ energy transfer process. The ^3^*H*_4_ state can be populated by the efficient nonradiative relaxation from the ^3^*F*_2,3_ state. The strong NIR UC (800 nm) is due to the ^3^*H*_4_ → ^3^*H*_6_ transition. In addition, the blue emission (476 nm) corresponds to the of ^1^*G*_4_ → ^3^*H*_6_ transition, where the ^1^*G*_4_ level is populated by the efficient energy transfer from the ^3^*H*_4_ state. The unexpected green emission (540 nm) is attributed to the co-doping of Tm^3+^/Er^3+^ ions in KMnF_3_ matrix. The resonant cross relaxation process ^3^*F*_4_ (Tm^3+^) + ^4^*F*_9/2_ (Er^3+^) → ^1^*G*_4_ (Tm^3+^) + ^4^*I*_15/2_ (Er^3+^) between Er^3+^ and Tm^3+^ ions leads to the population of ^1^*G*_4_ state of Tm^3+^ ions and depopulation of ^4^*F*_9/2_ state of Er^3+^ ions, and then to ^2^*H*_11/2_/^4^*S*_3/2_ state through the multiphonon assisted relaxations^29^.

## Conclusions

In summary, we have developed a facile hydrothermal method for preparation of tri-doped KMnF_3_ nanocubes with simultaneous four-color (blue, green, red and NIR) UC emissions. Of particular interests, the red and NIR bands, known as so-called “optical window” for imaging biological tissues, are strong. The spectral and pump dependence analyses indicate that two-photon process is responsible for the red and NIR emissions. We believe that this proof-of-concept demonstration of a multicolor emission across a broader spectra (blue to NIR) using tri-doped single KMnF_3_ host system may have potential applications for multiplexing analysis and/or multi-optical window imaging of biological tissues.

## Methods

### Materials

MnCl_2_, KF, KOH, ethanol, oleic acid (OA) at AR grade were obtained from Sinopharm Chemical Reagent Company, China. YbCl_3_·6H_2_O, ErCl_3_·6H_2_O, TmCl_3_·6H_2_O were obtained from CongHua City JianFeng Rare Earth Company, China. All other chemical agents obtained from commercial routes were of analytical grade and were used without further purification.

### Preparation of tri-doped KMnF_3_ nanocubes

The rare-earth tri-doped KMnF_3_ nanocubes were hydrothermally prepared by using MnCl_2_ and KF as precursors at 180 °C. Typically, 1.5 g (27 mmol) KOH, 2 mL H_2_O, 4 mL ethanol (48 mmol) and 9 mL of (24 mmol) OA (90 wt%) were well mixed at the room temperature for 10 min. A white viscous solution was obtained. The 10 mL (0.2 mol/L) MnCl_2_ solution, 15.5 mg (0.4 mmol) YbCl_3_·6H_2_O, 1.5 mg (0.04 mmol) ErCl_3_·6H_2_O and 1.5 mg (0.04 mmol) TmCl_3_·6H_2_O was subsequently added and vigorously stirred for 20 min. Then 8 mL (1.25 mol/L) KF was added into the above solution. After incubation for 1 h, the mixture was transferred to a 50 mL Teflon-lined autoclave, and then heated at 180 °C for 24 h. After cooling down, the products were removed by centrifugation then washed with ethanol, and dried under vacuum at room temperature for 24 h.

### Characterization

X-Ray powder diffraction (XRD) chracterization were carried out on a Rigaku D/max-*γ*B diffractometer equipped with a rotating anode and a Cu Kα source (λ = 0.15418 nm). SEM micrographs were obtained using a field emission scanning electron microscope (FESEM, MX2600FE). Upconversion luminescence spectra were measured by a regeneratively amplified 980 nm diode laser (Hi-Tech Optoelectronics Co. Ltd., Beijing). The emitted UC fluorescence signal was collected by a lens-coupled monochromator (Zolix Instruments Co. Ltd., Beijing) at 3 nm spectral resolution with an attached photomultiplier tube (Hamamatsu CR131). All measurements were performed at room temperature.

## Additional Information

**How to cite this article**: Wang, H. *et al.* Triple-doped KMnF_3_:Yb^3+^/Er^3+^/Tm^3+^ nanocubes: four-color upconversion emissions with strong red and near-infrared bands. *Sci. Rep.* **5**, 17088; doi: 10.1038/srep17088 (2015).
